# Association of pre-operative chronic kidney disease and acute kidney injury with in-hospital outcomes of emergency colorectal surgery: a cohort study

**DOI:** 10.1186/s13017-020-00303-6

**Published:** 2020-03-26

**Authors:** Katsunori Miyake, Masao Iwagami, Takayasu Ohtake, Hidekazu Moriya, Nao Kume, Takaaki Murata, Tomoki Nishida, Yasuhiro Mochida, Naoko Isogai, Kunihiro Ishioka, Rai Shimoyama, Sumi Hidaka, Hiroyuki Kashiwagi, Jun Kawachi, Hidemitsu Ogino, Shuzo Kobayashi

**Affiliations:** 1 Kidney Disease and Transplant Center, Shonan Kamakura General Hospital, Kanagawa, Japan; 2 Columbia Center for Translational Immunology, Columbia University Medical Center, New York, NY USA; 3 Department of Urology, Tokyo Women’s Medical University, Tokyo, Japan; 4 Department of Health Services Research, University of Tsukuba, Ibaraki, Japan; 5 Department of Non-Communicable Disease Epidemiology, London School of Hygiene and Tropical Medicine, London, UK; 6 Department of Surgery, Shonan Kamakura General Hospital, Kanagawa, Japan; 7 Narita Tomisato Tokushukai Hospital, Chiba, Japan

**Keywords:** Emergency surgery, Colorectal surgery, Chronic kidney disease, Acute kidney injury, End-stage renal disease

## Abstract

**Background:**

Pre-operative kidney function is known to be associated with surgical outcomes. However, in emergency surgery, the pre-operative kidney function may reflect chronic kidney disease (CKD) or acute kidney injury (AKI). We examined the association of pre-operative CKD and/or AKI with in-hospital outcomes of emergency colorectal surgery.

**Methods:**

We conducted a retrospective cohort study including adult patients undergoing emergency colorectal surgery in 38 Japanese hospitals between 2010 and 2017. We classified patients into five groups according to the pre-operative status of CKD (defined as baseline estimated glomerular filtration rate < 60 mL/min/1.73 m^2^ or recorded diagnosis of CKD), AKI (defined as admission serum creatinine value/baseline serum creatinine value ≥ 1.5), and end-stage renal disease (ESRD): (i) CKD(-)AKI(-), (ii) CKD(-)AKI(+), (iii) CKD(+)AKI(-), (iv) CKD(+)AKI(+), and (v) ESRD groups. The primary outcome was in-hospital mortality, while secondary outcomes included use of vasoactive drugs, mechanical ventilation, blood transfusion, post-operative renal replacement therapy, and length of hospital stay. We compared these outcomes among the five groups, followed by a multivariable logistic regression analysis for in-hospital mortality.

**Results:**

We identified 3002 patients with emergency colorectal surgery (mean age 70.3 ± 15.4 years, male 54.5%). The in-hospital mortality was 8.6% (169/1963), 23.8% (129/541), 15.3% (52/340), 28.8% (17/59), and 32.3% (32/99) for CKD(-)AKI(-), CKD(-)AKI(+), CKD(+)AKI(-), CKD(+)AKI(+), and ESRD, respectively. Other outcomes such as blood transfusion and post-operative renal replacement therapy showed similar trends. Compared to the CKD(-)AKI(-) group, the adjusted odds ratio (95% confidence interval) for in-hospital mortality was 2.54 (1.90–3.40), 1.29 (0.90–1.85), 2.86 (1.54–5.32), and 2.76 (1.55–4.93) for CKD(-)AKI(+), CKD(+)AKI(-), CKD(+)AKI(+), and ESRD groups, respectively. Stratified by baseline eGFR (> 90, 60–89, 30–59, and < 30 mL/min/1.73 m^2^) and AKI status, the crude in-hospital mortality and adjusted odds ratio increased in patients with baseline eGFR < 30 mL/min/1.73 m^2^ among patients without AKI, while these were constantly high regardless of baseline eGFR among patients with AKI. Additional analysis restricting to 2162 patients receiving the surgery on the day of hospital admission showed similar results.

**Conclusions:**

The differentiation of pre-operative CKD and AKI, especially the identification of AKI, is useful for risk stratification in patients undergoing emergency colorectal surgery.

## Background

Chronic kidney disease (CKD), defined as decreased kidney function and/or the presence of kidney damage [1, 2], is known to be associated with increased mortality and morbidity in both cardiac and non-cardiac surgery [3, 4]. In the National Surgical Quality Improvement Program database, pre-operative estimated glomerular filtration rate (eGFR) was significantly associated with increased risk of mortality and complication of major abdominal surgery, including colorectal surgery [5]. However, the study focused on elective surgery, hence, excluding emergency surgery [5].

The international consensus on acute kidney injury (AKI) was also established in the twentieth century [6]. Accordingly, there has been a large amount of evidence that post-operative incidence of AKI was associated with worse prognoses of cardiac and non-cardiac surgery [7–9]. However, most of the previous studies focused on post-operative AKI, instead of pre-operative AKI, in elective surgery [7–9]. This is probably because, in theory, the possibility of pre-operative AKI is none or extremely small in elective surgery.

Colorectal surgery is the most common type of major abdominal surgery [5]. If the lower gastrointestinal tract is blocked or perforated, colorectal surgery may be conducted as an emergency surgery; patients may have dehydration and sepsis pre-operatively, which are well-known risk factors for AKI [10]. Therefore, patients undergoing emergency colorectal surgery may also have pre-operative AKI, with or without baseline CKD. However, to our knowledge, there has been no study differentiating between pre-operative CKD and AKI status in emergency surgery.

Therefore, using the electronic health records of a large hospital chain in Japan, we examined the association of pre-operative CKD and/or AKI with in-hospital outcomes in patients with emergent colorectal surgery.

## Methods

### Data source

We conducted a retrospective cohort study, using the Tokushukai Medical Database [11]. The Tokushukai Group is a large Japanese hospital chain, managing over 70 hospitals across Japan, including 38 hospitals which participated in the Diagnosis Procedure Combination (DPC) system, a lump-sum payment system in Japan [12]. The Tokushukai Medical Database mainly consists of administrative claims data (namely, the DPC inpatient data) and electronic health records, including inpatient and outpatient blood test results. The DPC inpatient data include the following information: patients’ age and sex; admission and discharge dates; discharge status (dead or alive); main diagnosis, comorbidities at admission, and post-admission complications recorded by the attending physician using the 2003 version of the International Classification Disease 10th revision (ICD-10) codes [13]; types of surgery (coded with original codes and text data in Japanese); and drugs and procedures, including mechanical ventilation and renal replacement therapy (RRT), on a daily basis. In a previous validation study in the Japanese DPC inpatient data, sensitivity, specificity, positive predictive value, and negative predictive value of chronic renal failure diagnosis were 53.3%, 99.3%, 80.0%, and 97.7%, respectively [14].

The study was approved by the Tokushukai Group Joint Ethics Committee (reference number TGE00947-024) and conducted in adherence with the tenets of the Declaration of Helsinki. Informed consent from individual patients was waived because all data were anonymized for research purposes.

### Study population

The study population consisted of adult patients (aged 18 years or older) who were admitted to one of 38 hospitals (Additional file 1) participating in the Japanese DPC system in the Tokushukai Medical Database during the 7-year period between July 2010 and June 2017 and received emergency colorectal surgery within 3 days of admission. The relevant surgery was identified based on Japanese surgery codes: K639 (“surgery for acute pan-peritonitis”), K719 (“colectomy”), K726 or K736 (“colostomy”), and K740 (“rectal resection”). Because “surgery for acute pan-peritonitis” may be conducted not only for colorectal perforation but also for upper gastrointestinal perforation and cholecystitis/cholangitis, we excluded patients with both surgery codes K639 (“surgery for acute pan-peritonitis”) and K646–K689 suggesting upper abdominal surgery. If a patient was hospitalized and received the emergency colorectal surgery multiple times during the study period, we included only the first event into the analysis.

### Exposure

**Fig. 1:**
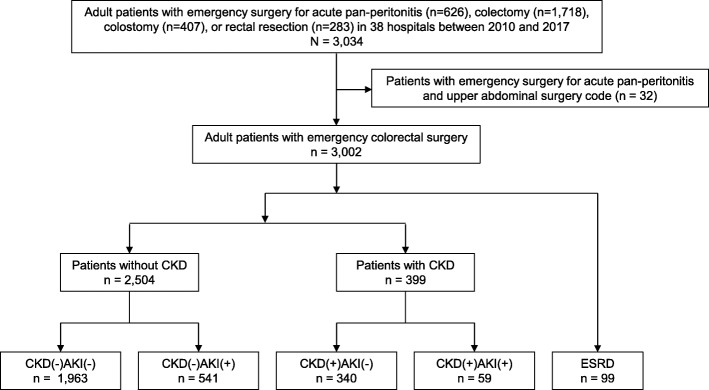
Flow chart of study participants selection. AKI, acute kidney injury; CKD, chronic kidney disease; ESRD, end-stage renal disease

The exposure of interest was pre-operative kidney function. For study purposes, we differentiated CKD, AKI, and end-stage renal disease (ESRD) using the following steps (Fig. 1). First, we identified patients with ESRD based on the admission diagnosis of ESRD (ICD-10 code N18.0 in the 2003 version of ICD-10 codes [13]) or the evidence of maintenance dialysis prior to the current admission, including hemodialysis and peritoneal dialysis. Next, we classified patients with and without CKD based on admission diagnosis of CKD (ICD-10 code N18.8 or N18.9 in the 2003 version of ICD-10 codes [13]) or baseline estimated glomerular filtration rate (eGFR) less than 60 ml/min/1.73 m^2^, according to the Kidney Disease Improving Global Outcomes (KDIGO) CKD criteria [2]. Baseline eGFR was calculated from the baseline serum creatinine value (the most recent value recorded within 7 to 365 days prior to current hospitalization) using the Modification of Diet in Renal Disease equation for Japanese patients [15]. Finally, in each group of patients with and without CKD, we identified patients with pre-operative AKI, defined as the ratio of serum creatinine value at admission and that at baseline ≥ 1.5, according to the KDIGO AKI criteria [6]. If the baseline serum creatinine value was not available in the database, we assumed that patients without CKD diagnosis had a GFR of 75 mL/min/1.73 m^2^ (according to the KDIGO AKI guideline [6]), and those with CKD diagnosis had the lowest serum creatinine value noted during current hospitalization [16]. Consequently, the study participants were grouped into the following five groups: (i) CKD(-)AKI(-), (ii) CKD(-)AKI(+), (iii) CKD(+)AKI(-), (iv) CKD(+)AKI(+), and (v) ESRD (Fig. 1).

### Outcomes

The primary outcome of interest was in-hospital mortality. Secondary outcomes included use of vasoactive drugs and mechanical ventilation on the next day of surgery or later (meaning that patients could not be weaned from them on the day of surgery); requirement for blood transfusion during hospitalization, including red cell concentrate, platelet concentrate, and fresh-frozen plasma; post-operative RRT, including intermittent and continuous RRT; and length of hospital stay among hospital survivors.

### Covariates

We considered the following baseline characteristics to examine the association between pre-operative CKD, AKI, or ESRD and the primary outcome (i.e., in-hospital mortality): age and sex; type of surgery based on the Japanese surgery codes (i.e., surgery for acute pan-peritonitis, colectomy, colostomy, or rectal resection); use of laparoscopy during operation; indication for surgery, including (i) peritonitis or perforation, (ii) obstruction, and (iii) bleeding or diverticulosis based on a list of ICD-10 codes shown in Additional file 2; presence or absence of colorectal cancer; body mass index (BMI); comorbidities, including diabetes, heart failure, chronic liver disease, chronic obstructive pulmonary disease, and cancer (except for colorectal cancer); and blood test results at admission, including white blood cell count, hemoglobin, platelet count, total protein, total bilirubin, and C-reactive protein. If a patient received multiple ICD-10 codes suggestive of an indication for surgery, (i) peritonitis or perforation was assigned the highest priority, followed by (ii) obstruction, and (iii) bleeding or diverticulosis, so that each patient was classified into only one of the three categories. A small number of patients with no ICD-10 code in the code list were categorized as “others”.

### Statistical analysis

First, we described the baseline patient characteristics by the five groups: (i) CKD(-)AKI(-), (ii) CKD(-)AKI(+), (iii) CKD(+)AKI(-), (iv) CKD(+)AKI(+), and (v) ESRD groups. Continuous variables (such as age and BMI) were presented as mean ± standard deviation (SD) and compared among the five groups by analysis of variance. If the distribution of the variable was skewed, we presented it as median (interquartile range [IQR]) and compared it among the five groups by Kruskal-Wallis test. Binary or categorical variables (such as sex and comorbidities) were presented as percentage and compared by chi-square tests.

Then, the study outcomes were presented and compared among the five groups, in the same way as the baseline characteristics. At subgroup analysis, we presented in-hospital mortality by baseline eGFR (> 90, 60–89, 30–59, and < 30 mL/min/1.73 m^2^) and AKI status, with 95% confidence intervals (CIs) estimated by binomial exact tests.

Finally, we conducted a multivariable logistic regression analysis to examine the independent association between the five groups and in-hospital mortality, adjusting for the aforementioned covariates and taking account of clustering by hospital using robust standard errors. We repeated the multivariable analysis according to the baseline eGFR (> 90, 60–89, 30–59, and < 30 mL/min/1.73 m^2^) and AKI status.

As post hoc analysis, we restricted the analyses to patients receiving the emergency surgery on the day of hospital admission, who were less likely to receive pre-operative management for kidney conditions than those receiving the surgery on the next day or later.

All statistical analyses were conducted using STATA version 14 (STATA Corp., Texas, USA).

## Results

We included 3002 eligible patients with emergency colorectal surgery (mean age 70.3 ± 15.4 years, male 54.5%) (Fig. 1). Among these, we identified 99 ESRD patients, including 82 patients with evidence of maintenance dialysis (80 with hemodialysis and 2 with peritoneal dialysis) and 17 patients with recorded diagnosis of ESRD at admission. We then identified 399 patients with CKD, including 369 patients with baseline eGFR < 60 mL/min/1.73 m^2^ and 30 patients with recorded diagnosis of CKD at admission. Finally, we identified 541 patients with AKI in patients without CKD and 59 in patients with CKD (Fig. 1).

Baseline characteristics are shown in Table 1. Although proportion of sex and type of surgery were not significantly different among the five groups, age distribution was significantly different: patients with CKD and/or AKI were apparently older than those in the CKD(-)AKI(-) and ESRD groups. Details of indication for surgery and proportion of colorectal cancer were also significantly different among the five groups. Notably, peritonitis or perforation was more common, while colorectal cancer was less common in patients with ESRD. Prevalence of diabetes and heart failure, as well as all the blood test results showed significant differences among the five groups. In particular, patients with CKD and ESRD showed lower hemoglobin levels, while patients with AKI showed higher C-reactive protein levels.

**Table 1:** Baseline characteristics of study participants with emergency colorectal surgery

	Total *n* = 3002	CKD(-)AKI(-) *n* = 1963	CKD(-)AKI(+) *n* = 541	CKD(+)AKI(-) *n* = 340	CKD(+)AKI(+) *n* = 59	ESRD *n* = 99	*P* value
Age (years), mean ± SD	70.3 ± 15.4	66.9 ± 16.1	76.8 ± 12.3	77.7 ± 10.8	78.9 ± 9.4	72.5 ± 10.8	< 0.001
Sex (male), %	54.5	45.5	46.8	44.7	47.5	40.4	0.815
Type of surgery, %							0.927
Surgery for acute pan-peritonitis	19.8	19.6	20.5	18.5	23.7	21.2	
Colectomy	57.2	56.8	58.4	57.4	57.6	58.6	
Colostomy	13.6	14.1	11.8	15.0	10.2	9.1	
Rectal resection	9.4	9.5	9.2	9.1	8.5	11.1	
Use of laparoscopy, %	4.0	5.0	1.5	3.2	0	1.0	0.001
Indication for surgery, %							< 0.001
Peritonitis or perforation	62.8	58.9	75.1	57.9	71.2	84.9	
Obstruction	25.3	27.4	18.3	30.3	23.7	5.1	
Bleeding or diverticulosis	2.3	2.5	0.9	3.8	1.7	1.0	
Others	9.6	11.2	5.7	7.9	3.4	9.1	
Presence of colorectal cancer, %	38.6	42.5	33.6	35.0	23.7	10.1	< 0.001
Body mass index (kg/m^2^), mean ± SD	21.3 ± 4.2	21.3 ± 4.2	21.5 ± 4.3	21.1 ± 3.8	21.6 ± 3.7	20.4 ± 4.2	0.108
Comorbidities, %							
Diabetes	14.4	12.2	14.6	21.8	20.3	27.3	< 0.001
Heart failure	4.0	2.5	4.4	10.6	3.4	9.1	< 0.001
Chronic pulmonary disease	3.8	4.0	3.9	3.8	1.7	2.0	0.786
Chronic liver disease	2.4	1.9	2.8	3.5	6.8	2.0	0.062
Cancer (except for colorectal cancer)	16.9	16.7	13.1	18.2	15.3	9.1	0.067
Serum creatinine tests^a^, median [IQR]							
Baseline creatinine (mg/dl)	0.75 [0.59–0.81]	0.72 [0.59–0.80]	0.72 [0.57–0.77]	1.07 [0.89–1.41]	1.01 [0.82–1.37]	-	< 0.001
Admission creatinine (mg/dl)	0.80 [0.64–1.07]	0.70 [0.59–0.83]	1.37 [1.14–1.90]	1.07 [0.83–1.44]	1.90 [1.56–2.84]	-	< 0.001
Blood tests at admission, median [IQR]							
White blood cell count (/1000 μl)	8.5 [5.0–12.5]	8.8 [5.4–12.8]	8.1 [4.4–12.8]	7.9 [5.07–10.8]	9.2 [5.1–12.5]	5.0 [2.8–9.7]	< 0.001
Hemoglobin (g/dl)	12.1 [10.0–13.8]	12.6 [10.5–14.0]	11.4 [9.6–13.6]	11.2 [9.4–12.9]	11.0 [8.8–12.6]	10.5 [9.0–11.7]	< 0.001
Platelet count (/10,000 μl)	21.5 [16.1–28.6]	22.7 [17.4–29.7]	19.7 [14.3–26.9]	19.7 [14.4–26.5]	17.0 [13.5–26.2]	15.0 [9.8–20.6]	< 0.001
Total protein (g/dl)	6.2 [5.1–7.0]	6.3 [5.3–7.0]	5.9 [4.6–6.8]	6.1 [5.1–6.9]	5.9 [4.9–6.8]	6.0 [5.2–6.7]	< 0.001
Total bilirubin (mg/dl)	0.7 [0.5–1.0]	0.7 [0.5–1.0]	0.8 [0.5–1.1]	0.7 [0.5–1.0]	0.7 [0.5–1.2]	0.4 [0.3–0.6]	< 0.001
C-reactive protein (mg/dl)	3.9 [0.5–14.2]	2.7 [0.3–11.9]	11.6 [2.3–22.0]	2.8 [0.5–12.7]	13.6 [3.4–21.8]	4.8 [1.0–16.0]	< 0.001

*AKI* acute kidney injury, *CKD* chronic kidney disease, *eGFR* estimated glomerular filtration rate, *ESRD* end-stage renal disease, *IQR* interquartile range, *SD* standard deviation

^a^Patients with ESRD were excluded

**Fig. 2:**
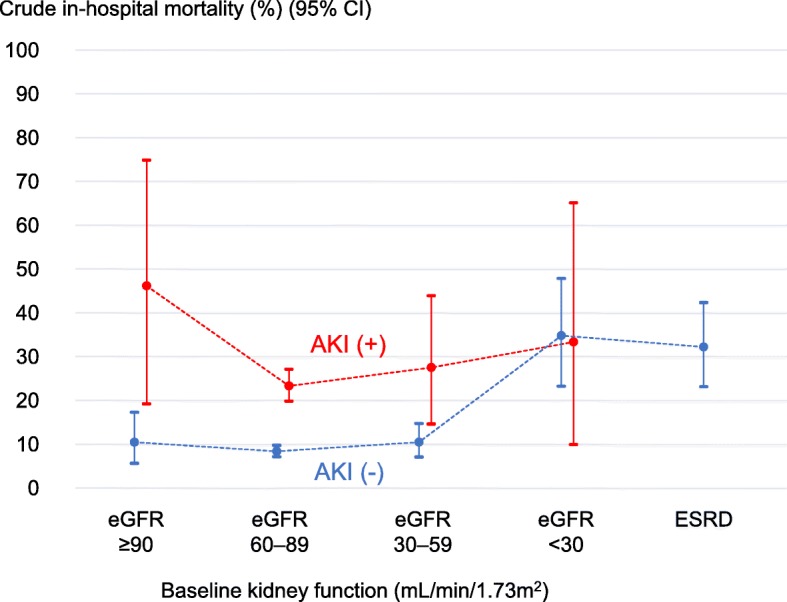
Crude in-hospital mortality by level of baseline kidney function and acute kidney injury status. CI, confidence interval; AKI, acute kidney injury; eGFR, estimated glomerular filtration rate; ESRD, end-stage renal disease

The in-hospital mortality was 8.6% (169/1963), 23.8% (129/541), 15.3% (52/340), 28.8% (17/59), and 32.3% (32/99) in the CKD(-)AKI(-), CKD(-)AKI(+), CKD(+)AKI(-), CKD(+)AKI(+), and ESRD groups, respectively (Table 2). In subgroup analysis, among patients without AKI, in-hospital mortality was around 10% in patients with baseline eGFR > 90, 60–89, and 30–59 mL/min/1.73 m^2^, whereas in-hospital mortality in patients with baseline eGFR < 30 mL/min/1.73 m^2^ and ESRD was over 30% (Fig. 2). Among patients with AKI, in-hospital mortality was over 20% regardless of baseline kidney function.

**Table 2:** In-hospital outcomes of study participants with emergency colorectal surgery

	Total *n* = 3002	CKD(-)AKI(-) *n* = 1963	CKD(-)AKI(+) *n* = 541	CKD(+)AKI(-) *n* = 340	CKD(+)AKI(+) *n* = 59	ESRD *n* = 99	*P* value
Primary outcome							
In-hospital mortality, %	13.3	8.6	23.8	15.3	28.8	32.3	< 0.001
Secondary outcomes							
Use of mechanical ventilation on the next day of surgery or later, %	26.9	21.1	43.8	24.7	44.1	46.5	< 0.001
Use of mechanical ventilation on the next day of surgery or later, %	6.5	4.0	10.7	8.2	15.3	21.2	< 0.001
Blood transfusion during hospitalization, %	38.6	30.2	56.8	46.8	55.9	68.7	< 0.001
Red cell concentrate, %	35.1	27.3	51.2	43.2	52.5	63.6	< 0.001
Platelet concentrate, %	4.7	3.0	8.3	4.1	10.2	16.2	< 0.001
Fresh-frozen plasma, %	24.3	18.0	41.0	25.0	39.0	45.5	< 0.001
Post-operative RRT, %	9.6	4.0	11.3	10.6	28.8	96.0	< 0.001
Continuous RRT, %	7.3	3.8	10.4	8.5	22.0	46.5	< 0.001
Intermittent RRT, %	4.2	0.4	2.0	2.9	11.9	90.9	< 0.001
Length of hospital stay in hospital survivors (days), median [IQR]	23 [14–40]	21 [13–35]	33 [20–55]	27 [16–42]	37 [18–58]	34 [24–69]	< 0.001

*AKI* acute kidney injury, *CKD* chronic kidney disease, *ESRD* end-stage renal disease, *IQR* interquartile range, *RRT* renal replacement therapy

Secondary outcomes, including vasopressors, mechanical ventilation, blood transfusion, post-operative RRT, and length of hospital stay showed similar trends with in-hospital mortality (Table 2). For example, the proportion of patients with any blood transfusion was 30.2%, 56.8%, 46.8%, 55.9%, and 68.7%, whereas post-operative RRT was 4.0%, 11.3%, 10.6%, 28.8%, and 96.0% in the CKD(-)AKI(-), CKD(-)AKI(+), CKD(+)AKI(-), CKD(+)AKI(+), and ESRD groups, respectively.

In multivariable logistic regression analysis, compared to the CKD(-)AKI(-) group, the adjusted odds ratio (95% CI) for in-hospital mortality was 2.54 (1.90–3.40), 1.29 (0.90–1.85), 2.86 (1.54–5.32), and 2.76 (1.55–4.93) in the CKD(-)AKI(+), CKD(+)AKI(-), CKD(+)AKI(+), and ESRD groups, respectively (Table 3). Among covariates, higher age, type of surgery, and cancer (except for colorectal cancer) were positively associated with increased risk of in-hospital death, whereas use of laparoscopy (probably because this factor suggests that patients were less severe), higher BMI, higher hemoglobin level, platelet count, and total protein level were negatively associated with in-hospital death. In subgroup analysis (Additional file 3), the shape of the association between baseline eGFR (> 90, 60–89, 30–59, and < 30 mL/min/1.73 m^2^) and AKI status, and in-hospital mortality was similar to that of the crude analysis: the adjusted odds ratio for in-hospital mortality increased in patients with baseline eGFR < 30 mL/min/1.73 m^2^ and ESRD among patients without AKI, while the adjusted odds ratio was constantly high regardless of the baseline eGFR among patients with AKI.

**Table 3:** Multivariable logistic regression analysis for in-hospital mortality

	Adjusted odds ratio (95% CI)	*P* value
Exposure groups		
(i) CKD(-)AKI(-)	1 (reference)	
(ii) CKD(-)AKI(+)	2.54 (1.90–3.40)	< 0.001
(iii) CKD(+)AKI(-)	1.29 (0.90–1.85)	0.166
(iv) CKD(+)AKI(+)	2.86 (1.54–5.32)	0.001
(v) ESRD	2.76 (1.55–4.93)	0.001
Covariates		
Age (per 1 year)	1.03 (1.02–1.05)	< 0.001
Sex (men vs. women)	1.39 (1.00–1.93)	0.051
Type of surgery		
Surgery for acute pan-peritonitis	1 (reference)	
Colectomy	1.67 (1.14–2.45)	0.009
Colostomy	2.40 (1.38–4.17)	0.002
Rectal resection	1.68 (0.98–2.87)	0.057
Use of laparoscopy (yes vs. no)	0.22 (0.07–0.71)	0.012
Indication for surgery		
Peritonitis or perforation	2.69 (0.55–13.13)	0.220
Obstruction	4.06 (0.93–17.80)	0.063
Bleeding or diverticulosis	1 (reference)	
Others	2.40 (0.51–11.23)	0.266
Presence of colorectal cancer (yes vs. no)	0.92 (0.62–1.35)	0.671
Body mass index (per 1 kg/m^2^)	0.94 (0.91–0.98)	0.001
Comorbidities (yes vs. no)		
Diabetes	1.24 (0.96–1.61)	0.095
Heart failure	1.66 (0.85–3.24)	0.135
Chronic pulmonary disease	1.14 (0.59–2.21)	0.699
Chronic liver disease	1.31 (0.71–2.40)	0.383
Cancer (except for colorectal cancer)	1.81 (1.32–2.46)	< 0.001
Blood test results at admission		
White blood cell count (per 1000/μl)	1.01 (0.98–1.04)	0.478
Hemoglobin (per 1 g/dl)	0.90 (0.85–0.95)	< 0.001
Platelet count (per 10,000/μl)	0.98 (0.96–0.99)	0.020
Total protein (per 1 g/dl)	0.88 (0.80–0.96)	0.005
Total bilirubin (per 1 mg/dl)	0.95 (0.74–1.23)	0.700
C-reactive protein (per 1 mg/dl)	1.00 (0.99–1.01)	0.588

*AKI* acute kidney injury, *CKD* chronic kidney disease, *CI* confidence interval, *eGFR* estimated glomerular filtration rate, *ESRD* end-stage renal disease

In post hoc analysis restricting to 2162 patients (72% of the study participants) receiving the emergency surgery on the day of hospital admission, the results were similar to those of the main analysis, with more prominent differences between the groups: the crude in-hospital mortality was 7.7% (108/1,396), 23.5% (101/430), 15.6% (35/224), 37.5% (15/40), and 29.2% (21/72) in the CKD(-)AKI(-), CKD(-)AKI(+), CKD(+)AKI(-), CKD(+)AKI(+), and ESRD groups, respectively, and the adjusted odds ratio (95% CI) for in-hospital mortality was 2.95 (2.05–4.25), 1.54 (0.88–2.70), 5.69 (2.52–12.84), and 2.87 (1.40–5.89) in the CKD(-)AKI(+), CKD(+)AKI(-), CKD(+)AKI(+), and ESRD groups, respectively, compared to the CKD(-)AKI(-) group.

## Discussion

Using a large database including over 3000 patients with emergency colorectal surgery in 38 community hospitals, we examined the association of pre-operative status of CKD and/or AKI with in-hospital outcomes. In crude analysis, the largest in-hospital mortality was observed in patients with ESRD (32.3%), followed by those with both CKD and AKI (28.8%), those with AKI only (23.8%), those with CKD only (15.3%), and those with neither CKD nor AKI (8.6%). In subgroup analysis, the in-hospital mortality increased from < 30 mL/min/1.73 m^2^ among patients without AKI, while the in-hospital mortality of patients with AKI was constantly high regardless of baseline kidney function. In adjusted analysis, CKD only was not significantly associated with increased mortality, but AKI only, AKI superimposed on CKD, and ESRD were independently associated with increased mortality.

Previous studies showed that there was an independent and graded association between CKD and poor patient outcomes, including cardiovascular events, hospitalization, and death [17, 18]. This was also found to be true in patients hospitalized for elective surgery: patients with renal dysfunction showed high post-operative mortality and morbidity [5, 19–21]. The worse prognosis in patients with decreased kidney function can be explained through a variety of mechanisms, including poor wound healing, post-operative infection due to decreased immunity, and coagulopathic disorders due to altered blood viscosity.

However, in emergency surgery, patients may also have pre-operative AKI, with or without baseline CKD. Potential reasons for AKI in emergency colorectal surgery include sepsis from peritonitis or bacterial translocation and renal ischemia from dehydration or bleeding [22]. Emergency operation can remove these underlying causes of AKI, and therefore, some people may expect that patients with pre-operative AKI would show better prognoses than those with pre-operative CKD. However, in our real-world data, patients in the CKD(-)AKI(+) group showed rather worse mortality and morbidity than those in the CKD(+)AKI(-) group and similar mortality with the ESRD group (Table 2). We speculate that patients with pre-operative AKI may be more likely to suffer from dysfunction of homeostasis, which are important for post-operative recovery [23].

Despite several studies showing an association between the post-operative incidence of AKI and worse mortality and morbidity in patients with elective surgery [24, 25], our study is the first to suggest the need for more attention for pre-operative AKI in the case of emergency surgery. In a previous study, some predictors (e.g., pre-operative sepsis or septic shock, number of comorbidities) of surgical mortality in patients with colorectal perforation were identified; however, the impact of pre-operative kidney function was not examined [22]. In addition to the current study indicating that pre-operative AKI is a good predictor for outcomes of emergency colorectal surgery, future studies are warranted to examine how clinicians, including surgeons, anesthesiologists, and nephrologists, could adopt a different approach to improve surgical outcomes in patients with and without pre-operative AKI. There may be some strategies to improve pre-operative kidney function in short pre-operative period (e.g., to postpone the surgery until urine output is established), while the majority (72%) of the study participants in our current study received the surgery immediately on the day of hospital admission.

We hereby acknowledge several limitations of the study. First, although our study findings were obtained from 38 community hospitals in Japan, its external validity may be limited to other Japanese hospitals (e.g., university hospital) and other countries. Notably, patients included in this Japanese study were slim, with mean BMI of 21.3 ± 4.2 kg/m^2^. The association between kidney function and outcomes of emergency colorectal surgery may be different in obese patients because obesity is associated with CKD [26], AKI [27], and surgical outcomes [28, 29]. Second, like most observational studies on kidney diseases, not all patients had their baseline kidney function measured in the community. We used the best available approach to classify pre-operative CKD and AKI, in line with the recent KDIGO CKD and AKI guidelines [2, 6]. However, misclassification of CKD and AKI status is still possible, and some patients in the CKD(-)AKI(-) group might have had CKD or AKI. If this was the case, the in-hospital mortality of patients in the CKD(-)AKI(-) group may have been overestimated, indicating that the true association between pre-operative CKD and/or AKI and in-hospital mortality may be larger than the adjusted odds ratios obtained in the current study. Third, there may be some unmeasured confounding factors in the association between kidney function and in-hospital outcomes. For example, we could not access operative notes in the current study; therefore, details of operation remained unknown. There may be some anesthesiologic and other measures that may increase the chance of better outcomes of emergent patients with AKI or CKD. It is also possible that complications due to surgical techniques or surgical decision-making caused post-operative AKI or directly influenced mortality and morbidity, but we were unable to take these into account in our analysis. In addition, it was difficult to define some surgical outcomes that we were originally interested in, such as wound infection and drain trouble, uniformly, in the 38 hospitals. Finally, although this study is one of the largest studies in this field, statistical power may still be limited in some analyses. A lack of independent association between CKD only and in-hospital mortality (adjusted odds ratio 1.29, 95% CI 0.90–1.85) may be possibly due to insufficient study power. In our subgroup analysis by baseline kidney function (Fig. 2 and Additional file 3), some groups showed wide CIs due to the small sample sizes. Further subgroup analysis, especially based on the AKI stage, was difficult.

As for clinical implications of the current study focusing on risk stratification, the study findings can be used to inform patients (and their family members) of their prognosis more accurately than before as part of informed consent. As for research implications, further research is warranted to investigate whether patient outcomes can be modified by directly intervening in the kidney function (e.g., postponing the surgery, with pre-operative fluid management, until urine output is established) or taking different treatment approach (e.g., aiming at different peri-operative arterial blood pressure) according to the pre-operative CKD and AKI status.

## Conclusions

This large study showed that the in-hospital outcomes of emergency colorectal surgery were significantly different according to the pre-operative status of CKD, AKI, and ESRD. CKD only was not significantly associated with increased mortality, but AKI only, AKI superimposed on CKD, and ESRD were independently associated with increased mortality. Thus, differentiation of pre-operative CKD and AKI is warranted for risk stratification in patients undergoing emergency colorectal surgery.

## Supplementary information

**Additional file 1.** List of 38 hospitals in the Tokushukai Medical Database.

**Additional file 2.** List of International Classification Disease of 10th revision codes to classify indication for surgery.

**Additional file 3.** Multivariable logistic regression analysis by level of baseline kidney function and acute kidney injury status. AKI = acute kidney injury, eGFR = estimated glomerular filtration rate, ESRD = end-stage renal disease.

## Data Availability

The data that support the findings of this study are available from the Tokushukai Group, Japan, but restrictions apply to the availability of these data, which were used under license for the current study, and so are not publicly available.

## Abbreviations

AKI: Acute kidney injury
BMI: Body mass index
CI: Confidence interval
CKD: Chronic kidney disease
DPC: Diagnosis Procedure Combination
eGFR: Estimated glomerular filtration rate
ESRD: End-stage renal disease
ICD-10: International Classification Disease 10th revision
IQR: Interquartile range
KDIGO: Kidney Disease Improving Global Outcomes
RRT: Renal replacement therapy
SD: Standard deviation
