# Supplementary material for: Association of pre-operative chronic kidney disease and acute kidney injury with in-hospital outcomes of emergency colorectal surgery: a cohort study
Source: World J Emerg Surg. 2020 Mar 26;15:22. doi: 10.1186/s13017-020-00303-6 (PMC7098074; doi:10.1186/s13017-020-00303-6)
Supplement: Supplementary file 1 — Additional file 1. List of 38 hospitals in the Tokushukai Medical Database. [file 13017_2020_303_MOESM1_ESM.pdf]

Additional file 1. List of 38 hospitals in the Tokushukai Medical Database

1. Sapporo Higashi Tokushukai Hospital	20. Uji Tokushukai Hospital
2. Sapporo Tokushukai Hospital	21. Kobe Tokushukai Hospital
3. Kyoaikai Hospital	22. Takasago Seibu Hospital
4. Shonai Amarume Hospital	23. Izumo Tokushukai Hospital
5. Koga Hospital	24. Nozaki Tokushukai Hospital
6. Tokyo-Nishi Tokushukai Hospital	25. Matsubara Tokushukai Hospital
7. Hanyu General Hospital	26. Kishiwada Tokushukai Hospital
8. Chiba Tokushukai Hospital	27. Yao Tokushukai General Hospital
9. Chiba-Nishi General Hospital	28. Uwajima Tokushukai Hospital
10. Kamagaya General Hospital	29. Fukuoka Tokushukai Hospital
11. Shonan Kamakura General Hospital	30. Nagasaki Kita Tokushukai Hospital
12. Shonan Fujisawa Tokushukai Hospital	31. Kagoshima Tokushukai Hospital
13. Hayama Heart Center	32. Osumikanoya Hospital
14. Shonan Atsugi Hospital	33. Yakushima Tokushukai Hospital
15. Shizuoka Tokushukai Hospital	34. Naze Tokushukai Hospital
16. Haibara General Hospital	35. Tokunoshima Tokushukai Hospital
17. Nagoya Tokushukai General Hospital	36. Chubu Tokushukai Hospital
18. Ogaki Tokushukai Hospital	37. Nanbu Tokushukai Hospital
19. Oumikusatsu Tokushukai Hospital	38. Miyakojima Tokushukai Hospital
