# Supplementary material for: Association of pre-operative chronic kidney disease and acute kidney injury with in-hospital outcomes of emergency colorectal surgery: a cohort study
Source: World J Emerg Surg. 2020 Mar 26;15:22. doi: 10.1186/s13017-020-00303-6 (PMC7098074; doi:10.1186/s13017-020-00303-6)
Supplement: Supplementary file 2 — Additional file 2. List of International Classification Disease of 10th revision codes to classify indication for surgery. [file 13017_2020_303_MOESM2_ESM.pdf]

Additional file 2. List of International Classification Disease of 10th revision codes to classify indication for surgery.

Indication for surgery	International Classification Disease of 10th revision codes
(i) Peritonitis or perforation	K35.2, K35.3, K40.1, K40.4, K41.1, K41.4, K42.1, K43.1, K43.4, K43.7, K44.1, K45.1, K46.1, K55, K57.0, K57.2, K57.4, K57.8, K63.1, K65
(ii) Obstruction	K40.0, K40.3, K41.0, K41.3, K42.0, K43.0, K43.3, K43.6, K44.0, K45.0, K46.0
(iii) Bleeding or diverticulosis	K57.1, K57.3, K57.5, K57.9, K92.1, K92.2
