# Supplementary figures and images for: Association of pre-operative chronic kidney disease and acute kidney injury with in-hospital outcomes of emergency colorectal surgery: a cohort study
Source: World J Emerg Surg. 2020 Mar 26;15:22. doi: 10.1186/s13017-020-00303-6 (PMC7098074; doi:10.1186/s13017-020-00303-6)

Adjusted odds ratio for in-hospital mortality (95% CI)

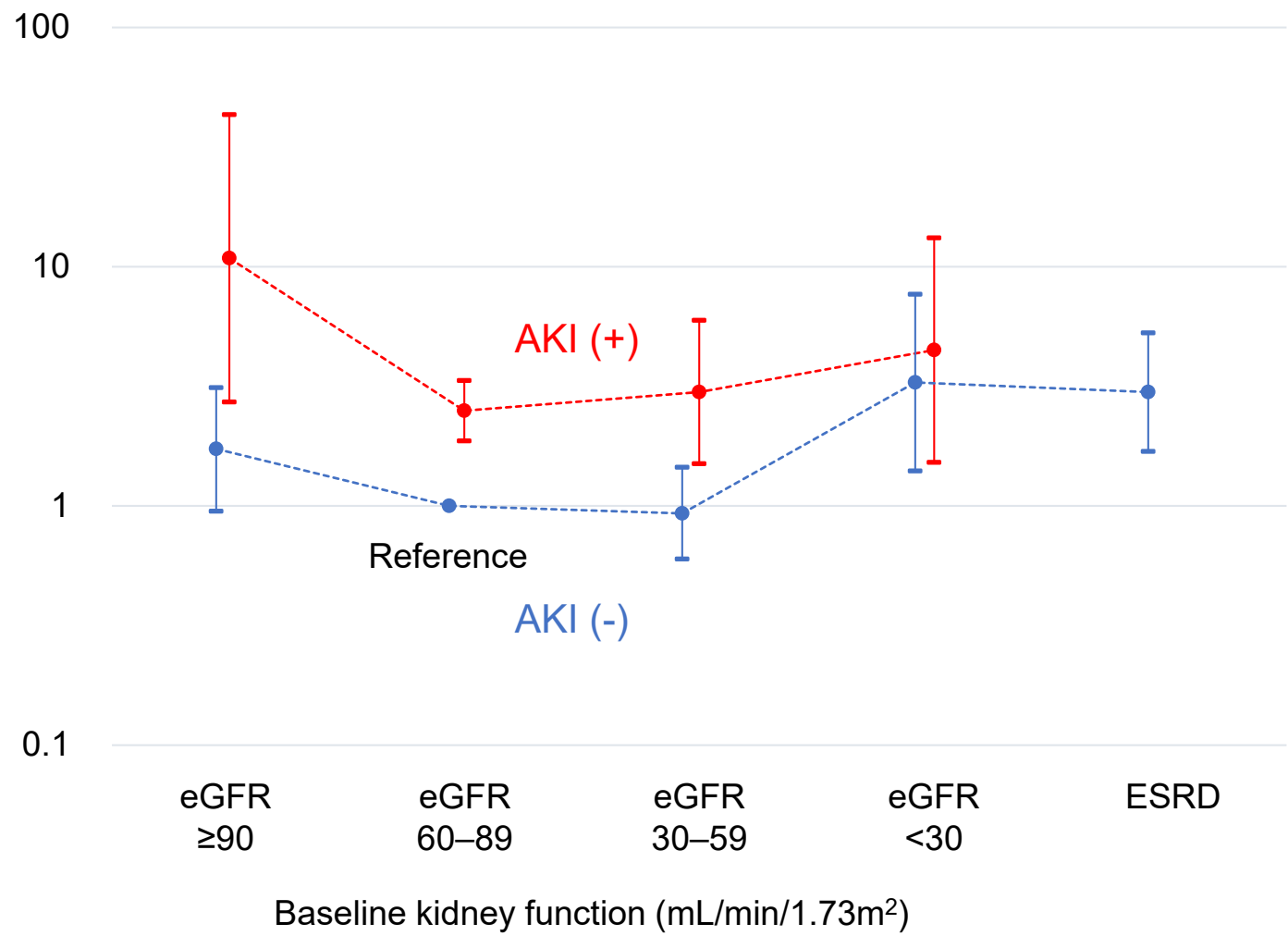

Supplement: Supplementary file 3 — Additional file 3. Multivariable logistic regression analysis by level of baseline kidney function and acute kidney injury status. AKI = acute kidney injury, eGFR = estimated glomerular filtration rate, ESRD = end-stage renal disease. [file 13017_2020_303_MOESM3_ESM.pdf]
